# A comparison of absorption and phase contrast for X-ray imaging of biological cells

**DOI:** 10.1107/S1600577518009566

**Published:** 2018-08-27

**Authors:** Colin Nave

**Affiliations:** a Diamond Light Source Ltd, Harwell Science and Innovation Campus, Didcot OX11 0DE, UK

**Keywords:** X-ray imaging, biological cells, dose and fluence requirements

## Abstract

X-ray imaging allows biological cells to be examined at a higher resolution than possible with visible light and without some of the preparation difficulties associated with electron microscopy of thick samples. The most used and developed technique is absorption contrast imaging in the water window which exploits the contrast between carbon and oxygen at an energy of around 500 eV. A variety of phase contrast techniques are also being developed. In general these operate at a higher energy, enabling thicker cells to be examined and, in some cases, can be combined with X-ray fluorescence imaging to locate specific metals. The various methods are based on the differences between the complex refractive indices of the cellular components and the surrounding cytosol or nucleosol, the fluids present in the cellular cytoplasm and nucleus. The refractive indices can be calculated from the atomic composition and density of the components. These in turn can be obtained from published measurements using techniques such as chemical analysis, scanning electron microscopy and X-ray imaging at selected energies. As examples, the refractive indices of heterochromatin, inner mitochondrial membranes, the neutral core of lipid droplets, starch granules, cytosol and nucleosol are calculated. The refractive index calculations enable the required doses and fluences to be obtained to provide images with sufficient statistical significance, for X-ray energies between 200 and 4000 eV. The statistical significance (*e.g.* the Rose criterion) for various requirements is discussed. The calculations reveal why some cellular components are more visible by absorption contrast and why much greater exposure times are required to see some cellular components. A comparison of phase contrast as a function of photon energy with absorption contrast in the water window is provided and it is shown that much higher doses are generally required for the phase contrast measurements. This particularly applies to those components with a high carbon content but with a mass density similar to the surrounding cytosol or nucleosol. The results provide guidance for the most appropriate conditions for X-ray imaging of individual cellular components within cells of various thicknesses.

## Abstract 2

The fluence and dose requirements as well as the expected contrast are calculated for X-ray imaging of selected cellular components as a function of photon energy. It is found that a much higher dose is required for phase contrast imaging compared with absorption contrast imaging in the water window.

## 1. Introduction   

X-ray imaging of biological cells allows higher resolution to be obtained compared with light microscopy and thicker specimens to be examined compared with electron microscopy. Radiation damage is an ultimate limitation using X-rays. In addition, long exposure times are required with some techniques, limiting their application for surveying variation between cells and imaging throughout the cell life cycle. It is therefore important to optimize the conditions for X-ray imaging and this paper gives some guidance as to how this could be done. It is assumed that 3D imaging is required in order to locate crowded organelles and macromolecules within a larger cell and that cryo techniques are used to minimize radiation damage while preserving the sample in as close to a native state as possible.

A good introduction to X-ray microscopy is given by Kirz *et al.* (1995 ▸). The various X-ray imaging techniques are based on either absorption or phase contrast and are briefly reviewed below.

### 1.1. Absorption contrast   

Absorption contrast instruments record differences in the transmission of X-rays as they pass through the sample. They are dependent on the differences in the imaginary part (β) of the refractive index of the cellular components. Consideration of the absorption edges of oxygen and carbon has led to the construction of successful full-field soft X-ray tomographic microscopes with zone plate objectives. These operate in the water window, where a high absorption contrast is expected between the carbon containing protein/lipid/nucleic acid components and the oxygen-rich water (Carrascosa *et al.*, 2009 ▸; Le Gros *et al.*, 2014 ▸, 2016 ▸; Duke *et al.*, 2014 ▸; Carzaniga *et al.*, 2013 ▸; Müller *et al.*, 2012 ▸;. Johansson *et al.*, 2004 ▸; Do *et al.*, 2015 ▸). These microscopes have revealed rich details about the organelles and virus particles contained within the cell. They have become a standard instrument used by many cell biologists.

The X-ray contrast for cellular organelles in the water window is due to the carbon-rich nature of the lipid membranes and embedded protein surrounding the organelles. These membranes have a thickness of 3–4 nm (approximately a factor of ten less than the resolution of current full-field X-ray microscopes) but perhaps up to 10 nm when embedded protein molecules (and sugars) are included. When viewed along or nearly along the plane of the membrane, there is a large amount of material in projection and this gives the high absorption contrast observed for the boundaries of cellular organelles. The advantages of collecting data in the water window from such samples are clear. The disadvantage is that X-ray absorption limits the thickness of material that can be examined.

### 1.2. Phase contrast   

Techniques that depend on measuring X-rays that are refracted or scattered away from the direct beam are often called phase contrast techniques. The phase shifts introduced by objects with different refractive indices normally dominate and depend on differences between the real parts (δ) of the refractive indices. The differences in the imaginary parts (β) of the refractive indices are usually much smaller than differences in δ and give amplitude shifts. These can become significant at energies in the region of absorption edges. Both components contribute to the complex electron density and have to be taken into account when calculating the number of photons scattered into the detector (Howells *et al.*, 2009 ▸). There are many variations of the phase contrast technique, including some that can independently measure the two components of the refractive index.

In propagation-based phase imaging (Snigirev *et al.*, 1995 ▸; Wilkins *et al.*, 1996 ▸; Cloetens *et al.*, 1996 ▸, 1999 ▸) X-rays are focused to a small focal spot, or emanate from a small source (Mayo *et al.*, 2003 ▸), and the specimen is placed downstream of the divergent X-rays so that it is fully illuminated. A detector placed some distance away records the transmitted X-rays. If the detector is near the sample, an absorption image is obtained. As the detector distance in increased, interference between the scattered and transmitted X-rays occur resulting in a hologram. The visibility and details of the resultant fringes depend on the detector position. Various methods are available for reconstructing the wavefront to obtain quantitative information. The resolution is limited by the size of the X-ray source or focal spot. The method has been used to obtain images of bacterial cells at cryotemperature (Bartels *et al.*, 2012 ▸) and room temperature (Bartels *et al.*, 2015 ▸).

In a Zernike phase contrast microscope (Vartiainen *et al.*, 2014 ▸), a zone plate objective is used and a phase plate inserted after the objective introduces a phase shift in the transmitted X-rays. At the detector, interference occurs between the transmitted X-rays and those scattered by the specimen giving a higher contrast for weakly absorbing specimens.

In X-ray grating interferometry (Weitkamp *et al.*, 2004 ▸; McDonald *et al.*, 2009 ▸) one or two gratings (one-dimensional or two-dimensional) are used to convert the high-frequency interference patterns produced by phase contrast into intensity modulations at the detector. The gratings can allow recording of scattering features which are much smaller than the detector pixel size. Quantitative information on absorption and phase shift can be obtained (*e.g.* by stepping the gratings).

In coherent diffraction imaging (CDI; Miao *et al.*, 1999 ▸), an isolated sample is fully illuminated with a coherent beam and the far-field diffraction pattern recorded on a suitable detector. Phase retrieval algorithms are used to recover an image of the sample. The method is being used on free-electron lasers for imaging reproducible particles such as large viruses (Ekeberg *et al.*, 2015 ▸).

In far-field ptychography (Rodenburg *et al.*, 2007 ▸; Thibault *et al.*, 2008 ▸; Rose *et al.*, 2015 ▸; Jones *et al.*, 2014 ▸; Rodriguez *et al.*, 2015 ▸; Diaz *et al.*, 2015 ▸, 2016 ▸; Deng *et al.*, 2015 ▸, 2017 ▸) a focused beam with a high degree of coherence is scanned across the specimen and the far-field scattered X-rays are recorded on a detector at each position of the incident beam. The step size is smaller than the focal spot size and the resulting overlap enables reconstruction of both the illumination and the complex refractive indices of the specimen. Compared with coherent diffraction imaging, the method allows the examination of extended specimens and appears to have supplanted CDI on synchrotron-based instruments. Far-field ptychography is very compatible with fluorescence imaging for identification of metals in cells (*e.g.* Deng *et al.*, 2017 ▸). The ability to obtain both the real and imaginary parts of the refractive index can also be used to identify high concentrations of metals within cells (*e.g.* Maiden *et al.*, 2013 ▸).

A variation of this is near-field ptychography (Stockmar *et al.*, 2015 ▸) in which the coherent X-rays are focused to a small spot and the specimen is placed downstream of the divergent beam, which fills the detector but not the specimen. The specimen is scanned as in far-field ptychography. This technique (called in this case ptychographic Fresnel coherent diffraction imaging) has been applied to imaging red blood cells infected with the malarial parasite (Jones *et al.*, 2013 ▸) to obtain a resolution of 70 nm in 3D. The method is less compatible with fluorescence imaging due to the larger beam size at the sample.

### 1.3. Comparison of absorption and phase contrast   

The phase contrast techniques provide the possibility of obtaining quantitative values for the real and imaginary parts of the refractive indices of the cellular components although multiple images (*e.g.* at different distances, with different phase rings, different grating positions) may be required. Absorption contrast techniques can provide information about the linear absorption coefficient (LAC) from which the imaginary part of the refractive index can be obtained. Zone plate objective lenses give a direct magnified image of the object but can have resolution, efficiency and depth of focus limitations. Coherent imaging techniques have the potential to circumvent these limitations but can themselves be limited by errors in phase retrieval, particularly for low-contrast weakly scattering samples.

Full-field soft X-ray absorption microscopy and coherent diffraction imaging were reviewed some years ago (Larabell & Nugent, 2010 ▸). The review recognized the different levels of maturity of the methods and contained the statement regarding coherent diffraction methods that ‘none of these images shows the vast array of subcellular organelles that are seen in images of *S. cerevisiae* obtained with transmission electron tomography or X-ray tomography’. Phase contrast methods including ptychography have not yet given resolutions significantly better than 100 nm in 3D (Rodriguez *et al.*, 2015 ▸) for biological cells with organelles of normal density. Sub-20 nm resolution has been obtained in 2D (Deng *et al.*, 2017 ▸) although this was obtained with a phase shift of approximately 0.7 rad, corresponding to a much thicker projection. Ptychography has achieved resolutions of 17 nm in 3D for harder materials (Holler *et al.*, 2014 ▸) and produced impressive 3D images of larger biological objects at around 100 nm resolution (Shahmoradian *et al.*, 2017 ▸).

The phase contrast based techniques have a common requirement to have sufficient photons to be scattered from one voxel of interest in the sample compared with the scattered photons in the surrounding material (*e.g.* cytosol or nucleosol in the case of a biological cell). Absorption contrast requires measurement of the difference between the photons transmitted by the voxel of interest and those transmitted by the surrounding material. In both cases adequate statistical significance is required and this has to be obtained before resolution damage destroys the specimen at the required resolution. This issue was examined in detail by Howells *et al.* (2009 ▸) for phase contrast imaging and the conclusion was that, with certain assumptions and requirements, a resolution of 10 nm could be achievable. This might be sufficient to identify larger protein complexes but such resolutions have not in any case been achieved in 3D. This paper attempts to address the contrast expected for biological cells at current resolutions where individual protein molecules are not expected to be resolved. It provides a reason why 3D resolutions of low-contrast cellular components in phase contrast microscopy, comparable with those obtained by absorption contrast microscopy, have not yet been obtained.

### 1.4. Overview of calculations   

Instead of calculating the contrast between protein and water it is assumed that the cellular interior (cytoplasm and nucleoplasm) contains a solution (cytosol and nucleosol) of molecules which give a baseline density. The contrast expected between this and four representative cellular components is calculated as a function of the X-ray energy. The components are inner mitochondrial membranes, heterochromatin, lipid droplet neutral core and starch granules. Values for refractive indices, densities and composition of some cellular components are available from electron microscopy and X-ray imaging at particular X-ray energies. By using these values, together with the calculated compositions, it is possible to calculate the expected contrast as a function of X-ray energy and, for example, reconcile measurements made in the water window and those at higher X-ray energies. The complex refractive index of a cellular component at a particular wavelength depends on both its density and chemical composition. Measurements at different energies provide additional information to separate out these factors and therefore can assist in identifying the cellular component as well as giving guidance on the most suitable energy to maximize the contrast of particular components before radiation damage occurs. The calculations allow comparison of absorption contrast and phase contrast methods and illustrate why some components have more visibility by phase contrast and others by absorption contrast.

The standard method for obtaining the contrast for X-ray imaging of biological cells has been based on calculating the real (δ) and/or imaginary (β) refractive indices for protein and water. The dose and flux required to image a feature (*e.g.* a protein molecule of 10 nm size) has been calculated in several publications (Howells *et al.*, 2009 ▸; Villanueva-Perez *et al.*, 2016 ▸; Starodub *et al.*, 2007 ▸; Shen *et al.*, 2004 ▸; Hagemann & Salditt, 2017 ▸; Jahn *et al.*, 2017 ▸). Some of these calculations are re­peated and the different assumptions and requirements of the various approaches are discussed. Calculations are then carried out for the cellular components considered here.

As there is a large variation in the composition of biological cells it is hoped that the results described in this paper will be refined with increasing data from X-ray imaging and other techniques and lead to a better understating of both the prospects for and results from X-ray imaging. Many of the errors present in actual data collection and analysis are ignored so that the approach represents a best-case scenario. It is hoped that this will be of value in identifying where effort is required to improve X-ray imaging procedures.

The ability to identify various cellular components depends on their dimensions together with the resolution of the imaging system. Table 2 in Appendix *A* [App appa] gives the approximate dimensions of some objects relevant to cell biology.

## 2. Calculation of contrast   

For X-ray imaging the resolution attainable is often inadequate to distinguish the various components. For example, even at the most optimistic resolution for X-ray imaging it would not be possible to distinguish between the nucleic acid and histone components in a nucleosome and even the putative 30 nm fibre for condensed chromatin would have to be treated as a single object at the resolutions currently obtainable for 3D imaging. Similarly, lipid membranes with smaller embedded proteins would have to be treated as a single object. An estimate therefore has to be made of the composition of the components, including bound water.

In order to calculate the real and complex refractive indices of objects visible for X-ray imaging, values are needed for their atomic composition and density. Information about the various components (*e.g.* lipids, proteins nucleic acids, water) is tabulated in Table 3 of Appendix *A* [App appa]. For each object to be imaged (*e.g.* mitochondrial membrane) the atomic compositions and densities of each component (*e.g.* lipid, protein) are combined (Table 4 of Appendix *A* [App appa]).

The contrast expected from X-ray imaging can then be calculated as a function of X-ray energy. The CXRO website provides the necessary tools for doing this (Henke *et al.*, 1993 ▸). For phase contrast, both the real (δ) and imaginary (β) parts of the complex refractive index contribute although, away from absorption edges, the real part dominates. For absorption contrast, the imaginary part contributes. The modulus of the electron density can be obtained from the expression
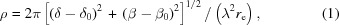
where the subscript zero refers to the background material (nucleosol or cytosol), λ is the wavelength and *r*_e_ is the classical radius of the electron.

The phase shift for a thickness *d* is given by
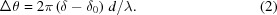
The estimates of the contrast do not apply in the immediate vicinity of absorption edges where, particularly for the light elements, complex behaviour occurs depending on the precise chemical environment.

## 3. Fluence, dose and the application of Rose criterion   

Knowledge of the composition and density of the cellular components allows calculations of the required fluence (photons µm^−2^) needed to obtain a defined resolution following the procedures for imaging a protein in water given by Howells *et al.* (2009 ▸) for phase contrast. Similar calculations can also be carried out for absorption contrast.

**Figure 1:**
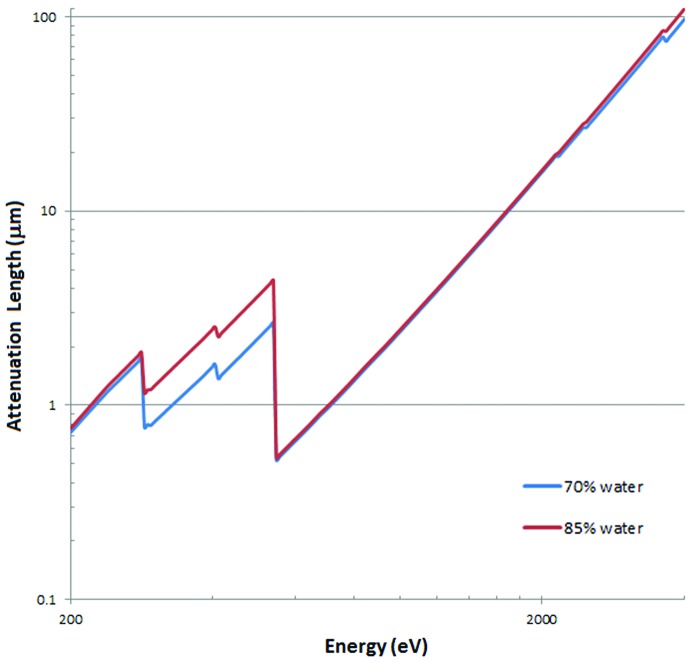
Whole cell attenuation lengths calculated for cells composed of 70% water and 85% water.

In Howells *et al.* (2009 ▸) the dose in Grays (Gy) required to obtain a particular contrast is calculated from the required fluence and the absorption properties of the particular cellular component. This calculation ignores the fact that the photo-electrons created by an absorbed photon can have a range bigger than the cellular component of interest. Approximate ranges in water are 20 nm at an electron energy of 200 eV and 400 nm at 4000 eV (Plante & Cucinotta, 2009 ▸). With uniform illumination of the cell, the dose deposited by photoelectrons is therefore likely to be distributed more evenly across the cell despite the fact that some components will absorb more incident X-rays. This option is illustrated for the case of protein in water and adopted for the dose calculations for imaging the cellular components. For thicker cells examined at low energy, the cell will not be uniformly illuminated (necessary for the Born approximation) as the X-rays will be attenuated along their path length as shown in Fig. 1. Highly absorbing lipid droplets for example might cluster together affecting the dose downstream. This affect would be less important for tomography where multiple views of the object are required. The attenuation does, however, provide a complication for tomographic reconstruction. A discussion of the application of the Born approximation is given by Kirz *et al.* (1995 ▸).

The issue of detecting an object within a noisy image was addressed by Rose (1948 ▸). He gave an estimate for the minimum detectable signal *k* of five times the r.m.s. noise based on studying television images of a test pattern. A separate calculation (Rose, 1973 ▸) used an example image which contained 10^5^ pixels each the area occupied by a test spot which had to be recognized as significant against the background. This gives 10^5^ opportunities of generating a false signal. It was also concluded that, in these circumstances, a signal with an amplitude *k* of five times the r.m.s noise was a reasonable estimate for satisfactory identification of an object. The Rose model was revisited by Burgess (1999 ▸) who covered its limitations. Despite these limitations, the adoption of the criterion (*k* = 5) for identifying an object has been widely adopted and can give useful estimates of detectability under defined circumstances as discussed by Burgess (1999 ▸).

For 3D imaging, it is the number of voxels, rather than the number of pixels, which should be considered when selecting the value of the Rose criterion *k*. With a volume of 10^5^ voxels, the Rose criterion would have to be increased to *k* = 6.8 to give the same probability of a false signal somewhere in the object as for a single isolated voxel with *k* = 5. However, only a modest increase in the required fluence (and dose) by a factor of 1.85 (6.8^2^/5^2^) would be required. In many cases the situation is somewhere between the two extremes. Planar objects (*e.g.* cell membranes) and linear objects (*e.g.* cellular filaments) lead to correlations between adjacent pixels thereby increasing the possibility of identification. In some cases the requirement might be to locate a particle (*e.g.* a virus) binding to these objects, in which case the relevant number of voxels is significantly reduced.

For CDI, the dependence of the dose and fluence requirements on the field of view (FOV) was also described by Villanueva-Perez *et al.* (2016 ▸). The FOV was defined in 1D as the linear dimension of the object (this defined the sampling requirement for the scattering pattern in that direction). For a two-dimensional projection the fluence requirements increased by (FOV/2σ)^2^ where 2σ is the feature size. The application of this correction to phase contrast techniques such as ptychography has not yet been evaluated. Villanueva-Perez *et al.* (2016 ▸) also show fluence graphs based on a value of *k* = 3 for CDI with the FOV correction.

The Rose criterion was used by Howells *et al.* (2009 ▸) and Villanueva-Perez *et al.* (2016 ▸) referring to the scattered intensity rather than the electron density of the object. There are two ways of doing this (Starodub *et al.*, 2007 ▸). In one approach the calculation is made for the total number of scattered photons into the detector from a single voxel in the object of size *d*/2 × *d*/2 × *d*/2 where *d* is the resolution. Alternatively, one can calculate the number of scattered photons from the entire object into one detector pixel at a scattering angle corresponding to the resolution of interest. The first method is not dependent on sample size whereas the second one is dependent on the size. Starodub *et al.* (2007 ▸) discussed the fact that the input to the numerical phase retrieval algorithms involves the modulus of the scattered amplitude rather than intensity. For Poisson noise, the conclusion was that a smaller number of scattered photons (6.25 rather than 25) would be required to satisfy the Rose criterion. This apparent discrepancy can be resolved by noting that the uncertainty in the density will follow the uncertainty in the amplitude which is itself a two-dimensional function of a complex variable. If the assumption is made that the phase error is small, then ρ/σ_ρ_ will be equal to 2*I*/σ_*I*_. Although this appears to give a different result from applying the Rose criterion to the intensity, the two can be reconciled by noting that the sampling interval for intensity measurements is half that for amplitude measurements. The required number of photons must fall into a so-called Shannon pixel that spans a solid angle just small enough to sample the diffraction pattern appropriately (Starodub *et al.*, 2007 ▸). The Shannon pixel for amplitude will have a solid angle four times that for intensity.

The Rose criterion can also be applied for imaging by absorption contrast. The signal for protein against water is
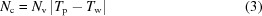
where *N*_v_ is the number of incident photons on a voxel, *T*_p_ is the transmission for protein and *T*_w_ is the transmission for water. The corresponding standard deviation is
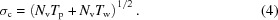
To observe the feature, we require *N*_c_ = *k*σ_c_ where *k* is the Rose criterion. This gives
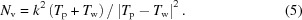
The required fluence is
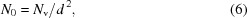
and the requuired dose (Howells *et al.*, 2009 ▸) is
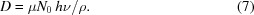
With absorption contrast and values of *T*_p_ and *T*_w_ near 1, the required number of photons (transmitted through a voxel) scales as *d* ^2^ due to the term |*T*_p_ − *T*_w_|^2^. The fluence and hence dose scales as the fourth power of the resolution. Hence the scaling with resolution in 3D is the same for both phase contrast and absorption contrast. In both cases the calculation of the required fluence and dose is dependent on the value of *k* adopted.

Villanueva-Perez *et al.* (2016 ▸) also compared projection microscopy (PM) in the holographic regime with CDI and concluded, for the parameters used, that the sensitivity of PM was a factor of approximately 2.3 (7/3) higher than CDI assuming no errors in the phase retrieval algorithms. They did, however, state that CDI has the potential to give better resolution provided the phase shifts can be resolved. Assuming the fluence requirement follows the square of the contrast, the increased contrast for PM would translate to a decrease in the fluence requirement of 5.3. Starodub *et al.* (2007 ▸) discussed the effects of error in the phase retrieval algorithms and showed (*e.g.* Fig. 7 of that paper) that the required fluence for a given resolution could be up to two orders of magnitude greater than estimates from the analytical procedures of Howells *et al.* (2009 ▸) and Shen *et al.* (2004 ▸). The fluence/dose requirements for absorption, near-field holography and CDI have also been addressed by Jahn *et al.* (2017 ▸) based on a maximum-likelihood approach. An extension of this work (Hagemann & Salditt, 2017 ▸) compared near-field holographic and far-field (CDI) imaging for a cell phantom taking into account errors in the phase determination step. In one of the examples in this paper, CDI required a factor of 37 (11000/300) more photons compared with near-field holography. The reduced performance of CDI was attributed to the reduced ability of the algorithms to decode the noisy diffraction patterns despite the similar information content for the two techniques considered. This analysis was carried out in 2D for a cell phantom with phase shifts differing by up to 1 rad, much higher than would be given by the components in a cell examined in 3D. As a result, the calculated fluences are much lower than the ones which are calculated in this paper. However, similar susceptibility of the reconstruction algorithm to noise might be expected for the lower phase shifts and correspondingly higher fluences discussed in this paper.

The efficiency of a zone plate should be taken into account if one is used as an objective for X-ray imaging. The overall efficiency is a combination of the attenuation of the zone plate and its modulation transfer function (MTF). Huang *et al.* (2009 ▸) used an overall efficiency of 2% for zone plate imaging when comparing a transmission X-ray microscope and CDI. Zone plates with a higher overall efficiency are being developed for harder X-rays (Mohacsi *et al.*, 2017 ▸).

The overall conclusion is that estimates of fluence and dose required to obtain a particular resolution are quite complex. A single value (*e.g.* the Rose criterion) has limited applicability particularly when applied at a pixel level (Burgess, 1999 ▸). However, the simple analysis is still useful when comparing required fluences and doses as a function of imaging methods and X-ray energy. In this paper, the analysis is given for protein against water following the approach of Howells *et al.* (2009 ▸) with additional analyses for the effects of field of view (for CDI), different Rose criteria, the effect of noise in a reconstruction algorithm (if used) and the efficiency of zone plate objectives (if used). A calculation of the dose assuming the deposited energy is spread throughout the cell rather than the component of interest is also included. The analysis for more complex cases (*e.g.* cellular organelles imaged against cytosol) is then given using the Howells *et al.* (2009 ▸) approach but assuming the energy from absorbed photons is deposited throughout the cell. Estimates for other requirements and assumptions can then be made by referring to the analyses for protein against water.

## 4. The atomic composition and density of cellular components   

The average values for the partial specific volume (PSV) of some of the principal components of conjugated proteins are 0.54, 0.61, 0.735 and 1.02 ml g^−1^, for the nucleic acid, carbohydrate, protein and lipid moieties, respectively (Durchschlag & Zipper, 1997 ▸). Values (Svergun & Koch, 2003 ▸) for the electron densities (e nm^−3^) are nucleic acids 550, proteins 420, lipids 300 and water 334. The values for lipids are not completely consistent between these two sources. Here it is assumed that the PSV of 1.02 ml g^−1^ for lipid refers to phospho­lipid and a value of 1.09 ml g^−1^ is used for lipid to give consistency with the electron density of 300 e nm^−3^.

Measured linear absorption coefficients (LAC) are available for some objects seen by full-field X-ray microscopy (Do *et al.*, 2015 ▸; Le Gros *et al.*, 2016 ▸; Weiss *et al.*, 2000 ▸), water content and atomic compositions from scanning electron microscopy (Nolin *et al.*, 2012 ▸, 2013 ▸) and density values from ptychography (Diaz *et al.*, 2015 ▸, 2016 ▸; Bartels *et al.*, 2012 ▸). These techniques have different resolutions and do not always give values which agree between the techniques, perhaps because a larger amount of water is included for the lower resolution techniques. One of the uncertainties in the analysis is the value for the density of water at cryogenic temperatures. Low-density amorphous ice formed from pure water has a density of 0.94 g ml^−1^. Both the cytosol and nucleosol contain relatively crowded biological and other molecules and it is not clear that this value will apply. In this paper, a density of 1.0 g ml^−1^ is used following Howells *et al.* (2009 ▸). This would lead to a slightly reduced contrast for many of the components at higher energies. However, the values of density and composition used for the cytosol and nucleosol give LAC values consistent with X-ray measurements (see §4.3 and §4.4). Values are summarized in Table 5 in Appendix *A* [App appa] and allow some validation of the composition and densities used for the calculations. A detailed calculation of the composition and density is given for one component (heterochromatin) as an example (Appendix *B* [App appb]).

### 4.1. Whole cell   

During X-ray imaging of cells, some of the X-rays are absorbed leading to a loss of transmitted signal. This will vary with the X-ray energy. A correction to various dose and fluence estimates for X-ray imaging can be obtained from the attenuation length which can be determined from the composition and density of the cell. The composition varies significantly between different types of cells and throughout the life cycle of a cell so only representative values can be used to give an estimate of the attenuation and the consequent loss of signal. The atomic composition of a cell was determined from the ratios 70% water, 8% nucleic acids, 16% protein, 2% lipids, 3% carbohydrates and 1% inorganic ions with associated metabolites (Watson, 1970 ▸) with potassium used to represent the inorganic ions. Within the water window, the attenuation length will be very dependent on the water content and a smaller value will be obtained for cells with a higher ratio of water to other components. Calculations are therefore also given for a cell with 85% water and other components reduced by a factor of two.

### 4.2. Heterochromatin (and ribosomes)   

A review of chromatin structure has been given by Maeshima *et al.* (2014 ▸).

Values for the mass ratios of nuclear chromatin are (Muramatsu & Onishi, 1978 ▸) DNA 1.0, RNA 0.11, acid soluble protein 1.27, alkaline soluble protein 0.95, giving an overall protein:nucleic acid ratio of 2:1. The packing density of condensed chromatin in eukaryotic cells has been measured at 400 mg ml^−1^ (Bohrmann *et al.*, 1993 ▸). This value is in reasonable agreement with the water content which has been measured at 65% for condensed chromatin (Nolin *et al.*, 2012 ▸). The two components can be divided up as 133 mg nucleic acid and 267 mg protein according to the above mass ratios. The volume occupied by each component has to be calculated in order to obtain the water content and then the atomic composition and density calculated (see Appendix *B* [App appb]).

The LAC can be calculated from the atomic composition and density and gives a value of 0.5 µm^−1^ at 520 eV, compared with values of 0.25–0.36 µm^−1^ (Table 5, Appendix *A* [App appa]) obtained from soft X-ray microscopy (Do *et al.*, 2015 ▸; Le Gros *et al.*, 2016 ▸). Possible explanations are that a greater amount of water surrounding the chromatin is included in the measurement from the lower-resolution X-ray microscopy or a less condensed state was examined. The calculations in this paper therefore give a more optimistic estimate of the contrast for heterochromatin than would be expected from the measurements by X-ray microscopy in the water window. Much higher compaction of nucleic acids can occur in bacterial cell nucleoids where density values up to 1.65 g cm^−3^ have been determined (Bartels *et al.*, 2012 ▸).

Ribosomes have a range of nucleic acid to protein ratios (Melnikov *et al.*, 2012 ▸), with mitochondrial ribosomes having a similar nucleic acid to protein ratio to heterochromatin (Sharma *et al.*, 2003 ▸). The calculations for heterochromatin can also serve for these cellular components with the proviso that the water content could be significantly different.

### 4.3. Cytsol   

Numbers for the concentration of macromolecules in the cytoplasm can be up to 300–400 g l^−1^. Much of this is likely to be associated with membranes and other structures which are visible by X-ray imaging. At present resolutions for X-ray imaging, many smaller components are not visible and these components may be included with the cytosol. A figure of 90 g l^−1^ is adopted for the average cytosol concentration of macromolecules, split between protein (60 g l^−1^) and nucleic acid (30 g l^−1^). This ratio is consistent with the measured N/P ratio of 7.4 ± 1.7 in the cytosol from scanning electron microscopy. In the present paper, the nucleic acid is being used to represent other phospho­rous containing components in the cytosol.

The measured LAC values for the cytoplasm (Le Gros *et al.*, 2016 ▸) show a peak at a LAC value of 0.27 µm^−1^ with a shoulder extending to approximately 0.1 µm^−1^. The composition adopted here gives an attenuation length of 5.24 µm at 520 eV or a LAC of 0.19 µm^−1^. This LAC value is at the lower end of that found within cells by Do *et al.* (2015 ▸) and the water content (91% by mass) is also at the higher end of that found for cytosol in cells (Nolin *et al.*, 2013 ▸). This gives partial support to the adoption of this composition as a ‘background’ against which the contrast of other cellular objects can be calculated. The resultant calculated density of 1.03 g ml^−1^ is lower than the measured value of (1.07 g ml^−1^) given by Diaz *et al.* for cytoplasm (rather than cytosol). The resolution achieved by Diaz *et al.* was 180 nm for the frozen hydrated cell and it is possible that some larger macromolecular components were included in the measurements.

### 4.4. Nucleosol   

The LAC values for obtained for euchromatin (Do *et al.*, 2015 ▸) vary between 0.13 and 0.25 µm^−1^, a similar range to cytosol. It is assumed that euchromatin is dispersed throughout the nucleus and that the LAC value can serve for nucleosol. The same protein/nucleic acid composition and density was therefore adopted for nucleosol as for cytosol but with the addition of a potassium concentration of 180 mmol (7 g l^−1^).

### 4.5. Mitochondria inner membrane   

In mitochondria, the inner and outer membranes are characterized by different phospho­lipid compositions and protein-to-lipid ratios. For the outer membrane, this ratio is about 1:1, whereas for the inner membrane, the protein:lipid ratio is 4:1 (Hallermayer & Neupert, 1974 ▸).

The structure of the inner membrane is quite complex and consists of complex cristae with total widths of up to 28 nm (Frey & Mannella, 2000 ▸).

It is assumed that the 28 nm-thick cristae consist of phospho­lipids, integral membrane proteins, peripheral membrane proteins and water. A 28 nm-thick structure can be obtained with two 4 nm-thick bilayers, each consisting of equal volumes of buried membrane protein and phospho­lipid plus additional bound proteins and water in the mass ratios protein:lipid:water of 556:139:450 and a density of 1.145 g ml^−1^. The total thickness of the structure is within the range of X-ray microscopes operating in the water window.

### 4.6. Lipid droplets neutral core   

Reviews of lipid droplet structure are given by Fujimoto & Parton (2011 ▸) and Thiam *et al.* (2013 ▸).

For the calculations here, it is assumed that the lipid droplets’ neutral core consists of tri­acyl­glycerol with the formula C_55_H_98_O_6_. A value of 0.92 g ml^−1^ was used for the mass density giving an electron density of 0.3 e Å^−3^, a value commonly used for solution scattering calculations (Svergun & Koch, 2003 ▸). This composition and density gives a LAC value of 0.89 µm^−1^ at 520 eV. This is slightly higher than the upper value of 0.75 ± 0.09 µm^−1^ quoted by Uchida *et al.* (2009 ▸). The reason for this discrepancy is unclear but it is possible that the lipid droplets measured by Uchida *et al.* contained some amphiphilic molecules and accompanying water.

### 4.7. Starch   

The formula used for starch is C_6_H_10_O_5_ with a partial specific volume of 0.601 ml g^−1^ (Durchschlag & Zipper, 1997 ▸). The structure and density of starch varies with water content (Bogracheva *et al.*, 2002 ▸).

A water content of 38% was used, giving a density of 1.32 g ml^−1^, intermediate between the values for starch platelets and starch grains determined from X-ray ptychography (Diaz *et al.*, 2015 ▸).

## 5. Results   

### 5.1. Attenuation lengths of cells   

Fig. 1 ▸ gives the attenuation length as a function of X-ray energy of two example biological cells containing 70% and 85% water. The main difference in attenuation between the two example cells is within the water window where, at 520 eV, the attenuation lengths are 2.5 µm (70% water) and 4.1 µm (85% water). Much higher attenuation lengths are obtained above 2 keV for both cells.

### 5.2. Protein in water   

**Figure 2:**
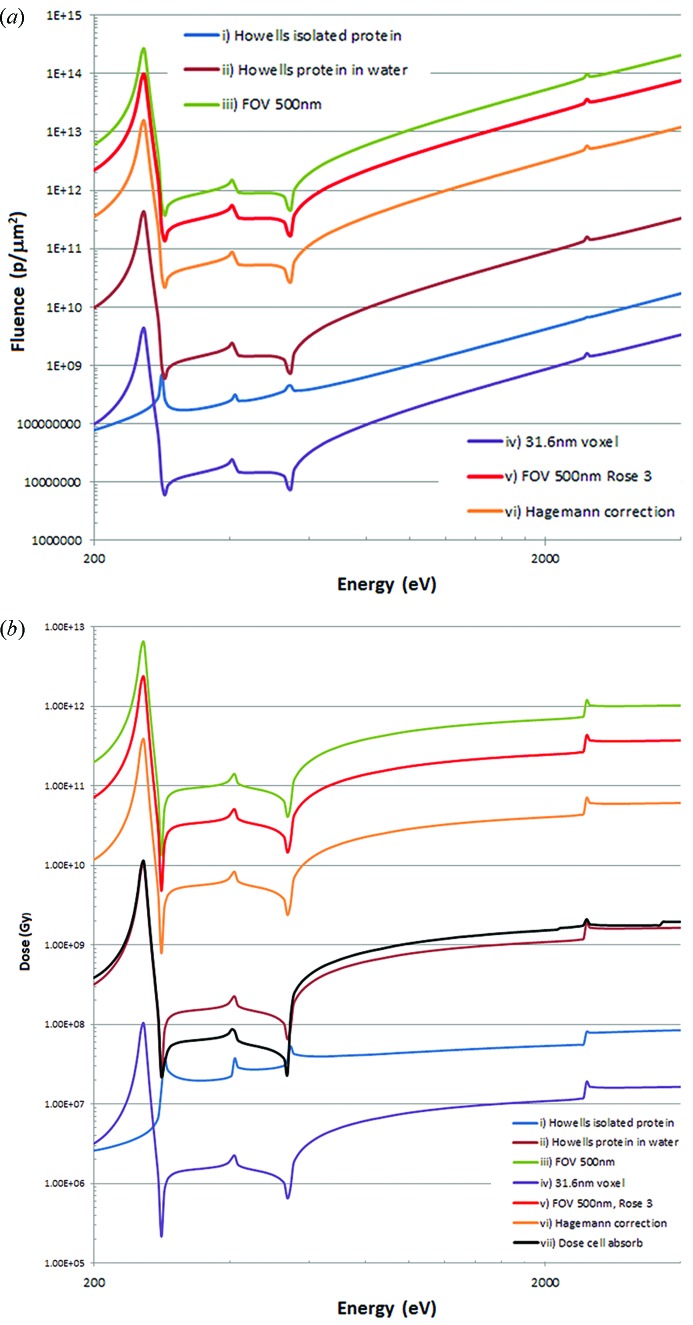
(*a*) Fluence to identify a protein with different requirements and assumptions. (i) Isolated protein in single 10 nm pixel and Rose criteria of 5 (Howells *et al.*, 2009 ▸). (ii) Protein in water, single 10 nm pixel (Howells *et al.*, 2009 ▸). (iii) As (ii) but within a field of view (FOV) for CDI of 500 nm (Villanueva-Perez *et al.*, 2016 ▸). (iv) As (ii) with a 31.6 nm voxel. (v) As (iii) but with a Rose criterion of 3. (vi) As (ii) but including errors in the phase retrieval algorithm (Hagemann & Salditt, 2017 ▸) giving a factor of 37 increase in required fluence. (*b*) Dose. (i)–(vi) as in (*a*). (vii) As in (ii) but with dose distributed over a model cell (70% water) rather than confined to a protein.

Figs. 2(*a*) and 2(*b*) ▸ show the fluence and dose calculations for imaging protein in vacuum and water at 10 nm resolution as calculated by Howells *et al.* (2009 ▸) based on CDI. Also shown are calculations applicable for a 500 nm field of view with a Rose signal-to-noise factor of 5 and 3 following Villanueva-Perez *et al.* (2016 ▸). When the feature to be imaged equals half the field of view (*e.g.* 10 nm in a 20 nm field of view), the two analyses give the same result. Calculations following Howells *et al.* (2009 ▸) with a 31.6 nm resolution, giving a 100-fold reduction in required dose and fluence, are also shown. The increased dose requirements due to errors in a CDI phase retrieval algorithm following the results of Hagemann & Salditt (2017 ▸) are shown to give some indication of the effects of these errors on the fluence and dose requirements, assuming that these results will apply to the lower contrast/higher fluence situation for the calculations in this paper. The other assumption is that this factor is simply applied as a correction for CDI and that the results for near-field holographic imaging and CDI are otherwise the same and similar to the Howell’s *et al.* analysis.

Finally, to illustrate the effect of cell absorption, the Howells *et al.* (2009 ▸) calculation is also shown for protein in water assuming that the dose is an average for a whole cell rather than that due to absorption by the protein alone. This shows lower dose requirements within the water window where the water component has a lower absorption coefficient but a higher dose outside the water window at higher energies due to the increased absorption of oxygen.

**Figure 3:**
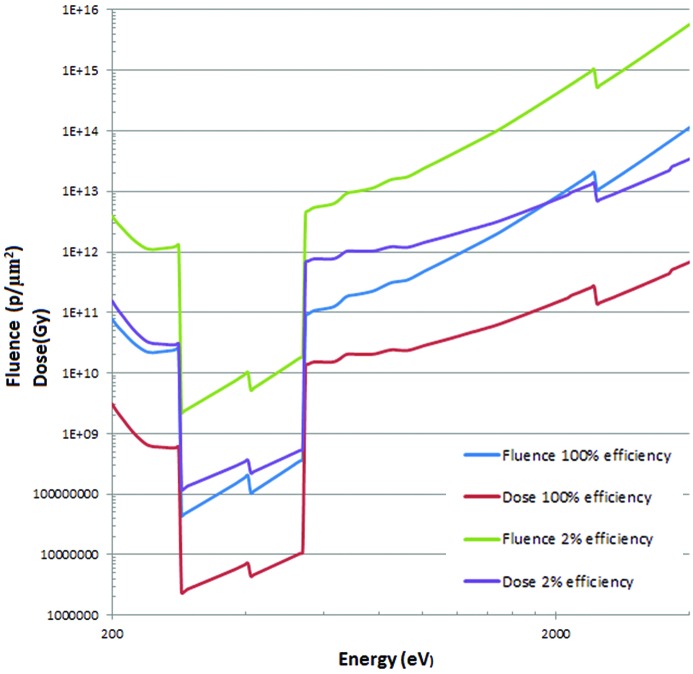
Fluence and dose for protein in water with absorption contrast. Rose criteria 5 with the dose distributed over a model cell (70% water). The fluence and dose at 2% efficiency follows the zone plate efficiency adopted by Huang *et al.* (2009 ▸).

Fig. 3 ▸ shows the fluence and dose curves for protein in water using absorption contrast. Included is the effect of limited efficiency of the zone plate if this is used. Modifications of these for the resolution and the dose model (for the protein itself or the whole cell) could be derived as in Figs. 2(*a*) and 2(*b*) ▸.

### 5.3. Refractive indices, dose and fluence for cellular components   

**Figure 4:**
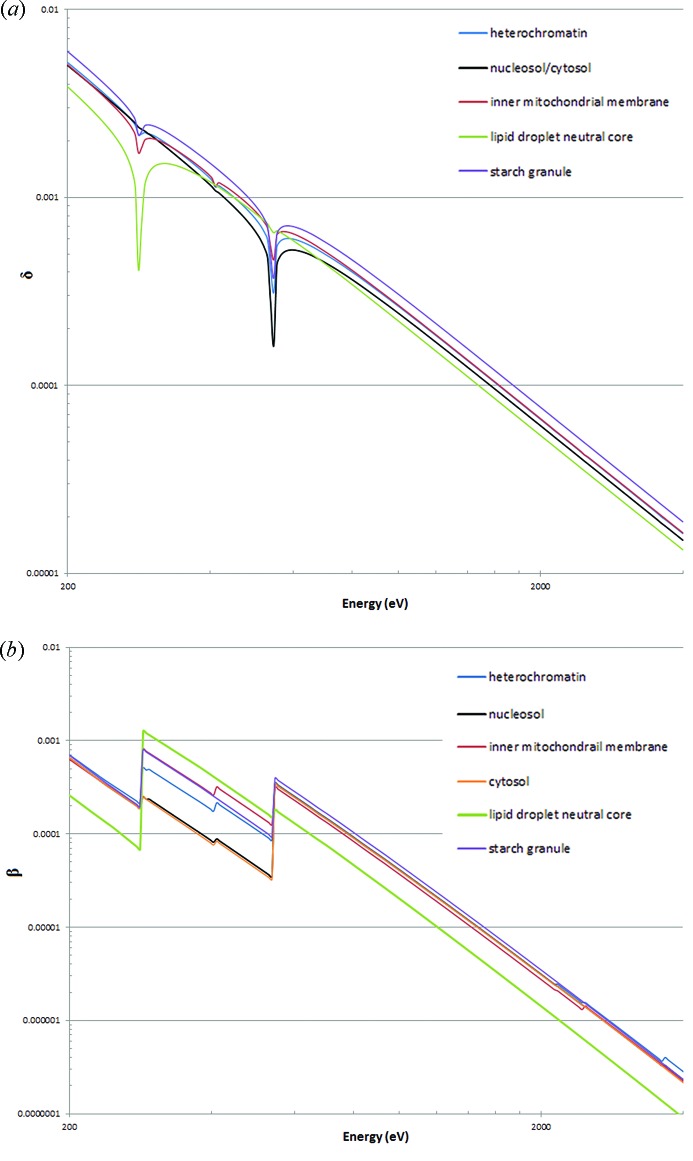
(*a*) The real part of the refractive index (δ) for the four cellular components together with cytosol and nucleosol, which are shown as a single line. (*b*) The imaginary part of the refractive index (β).

The refractive indices of the cellular components are shown in Figs. 4(*a*) and 4(*b*) ▸. As some of the curves are rather close together, values for the refractive indices at particular energies of interest are shown in Table 1 ▸. Changes in β above the phospho­rous, sulfur and potassium edges can be seen but these are much smaller than the changes seen across the water window.

**Figure 5:**
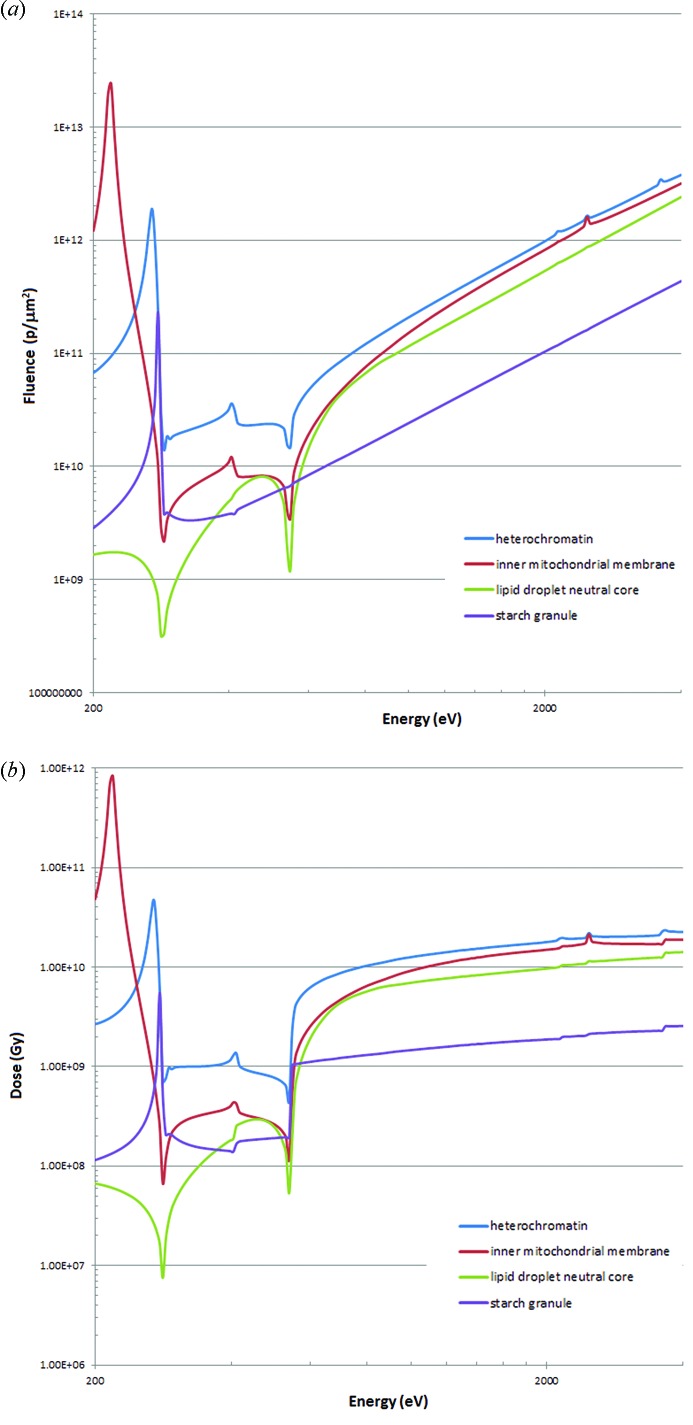
(*a*) Fluence requirements (phase contrast, 10 nm resolution) for the four cellular components following the calculations for protein illustrated in Fig. 2(*a*) ▸ curve (ii). (*b*) Dose requirements following the calculations in Fig. 2(*b*) ▸ curve (vii).

**Figure 6:**
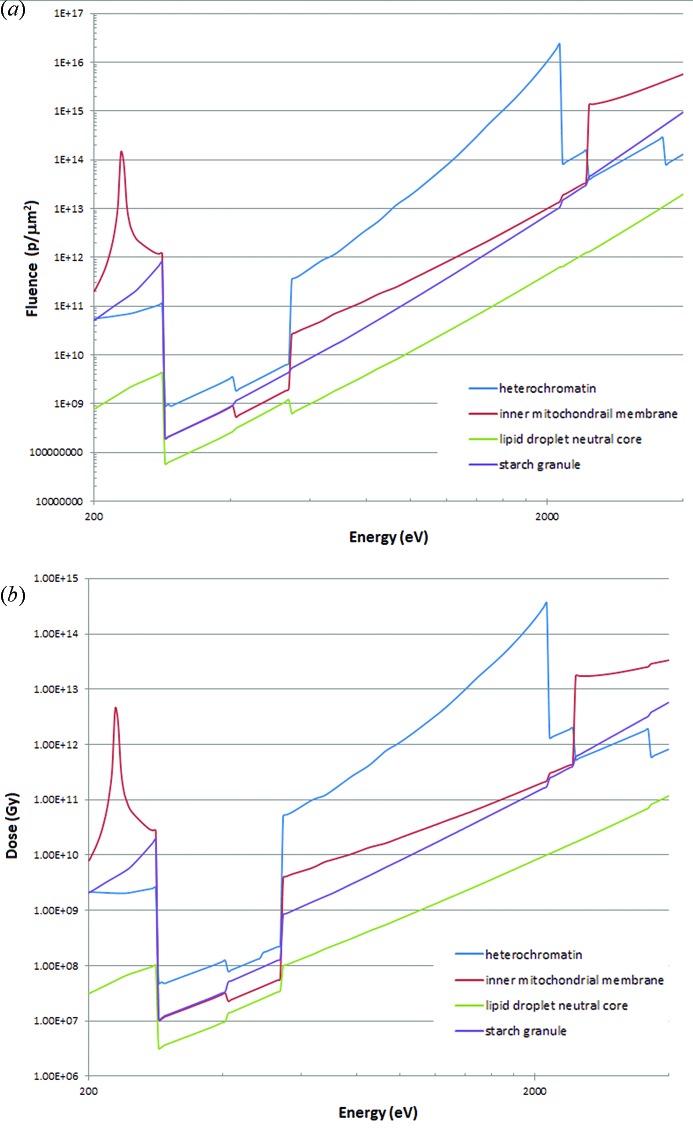
(*a*) Fluence requirements (absorption contrast, 10 nm resolution) for the four cellular components following the calculations for protein illustrated in Fig. 3 ▸, 100% efficiency. (*b*) Dose requirements following the calculations in Fig. 3 ▸, 100% efficiency.

The required fluences for achieving the Rose criterion based on the approach of Howells *et al.* (2009 ▸) for phase contrast are shown in Fig. 5(*a*) ▸. The doses, assuming the deposited energy absorbed by the cell [as in Fig. 2(*a*) ▸ for protein], rather than just the particular cellular component, is shown in Fig. 5(*b*) ▸. Data collection above the absorption edges for phospho­rous, sulfur and potassium gives small increases (rather than decreases) in the required dose. Corresponding graphs of fluence and dose for absorption contrast are shown in Figs. 6(*a*) and 6(*b*) ▸. The large changes in required fluence and dose seen above the phospho­rous, sulfur and potassium edges are not particularly relevant as they occur at high overall required dose and fluence, in the regime where phase contrast measurements are most appropriate. Modifications for other cases could be derived as in Figs. 2(*a*) and 2(*b*) ▸.

#### 5.3.1. Heterochromatin (and ribosomes)   

The refractive indices for the hetero chromatin are compared with those for nucleosol. Significant absorption contrast (β) is predicted within the water window although it is rather lower than for the other cellular components. The values of β for heterochromatin and nucleosol are almost identical between 550 eV and 2000 eV and little absorption contrast would be expected. There is a small increase in contrast above the potassium and phospho­rous absorption edges. However, in this energy range and above, phase contrast measurements are more likely to be carried out.

The fluence and dose curves are as expected with higher values compared with the other three cellular components both within the water window for absorption contrast and at higher energies for phase contrast.

#### 5.3.2. Mitochondrial inner membrane   

Mitochondrial inner membranes have a high positive absorption contrast within the water window. This is consistent with the ease with which mitochondria can be identified by absorption microscopy at around 520 eV. The phase contrast outside the water window is much lower, consistent with the statement (Larabell & Nugent, 2010 ▸) concerning the difficulty of observing many sub-cellular organelles by coherent diffraction techniques.

#### 5.3.3. Lipid droplets, neutral core   

Lipid droplets have a very high positive absorption contrast within the water window and a negative absorption contrast outside the water window. The phase contrast is much smaller and positive within the water window and negative outside the window. At about 700 eV, the value of δ for lipid droplets and cytosol is approximately the same (see Fig. 4*a* ▸) and the phase contrast is mainly due to the difference in β. This demonstrates that both phase and amplitude should be examined for techniques which can measure these separately. The required dose for absorption contrast and phase contrast are very similar at 2 keV.

Biological membranes with a small protein content would be expected to have intermediate contrast between those for lipid droplets and mitochondrial inner membranes.

#### 5.3.4. Starch granules   

Starch granules have a high absorption contrast within the water window and the highest phase contrast of the cellular components discussed here across most of the energy range, except in the immediate areas adjacent to absorption edges. This is largely due to the high electron density of these components. The required dose and fluence for both contrast mechanisms is correspondingly low. Even higher contrast would be expected for starch granules with lower water content and correspondingly higher mass density.

### 5.4. Comparison of dose for absorption and phase contrast   

**Figure 7:**
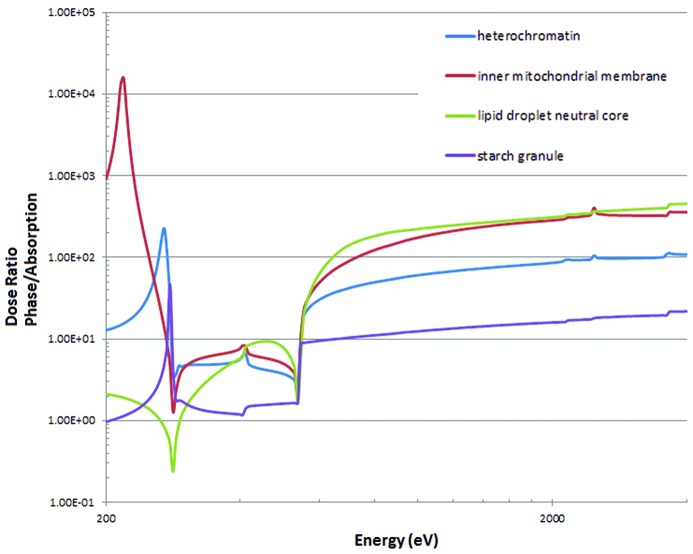
Comparison of the dose for phase contrast with absorption contrast at 520 eV. Obtained by dividing the values in Fig. 5(*b*) ▸ with the value at 520 eV in Fig. 6(*b*) ▸.

As most full-field soft X-ray imaging of cells is carried out at energies around 520 eV, it is of interest to compare the dose and fluence requirements at this energy with those for phase contrast at higher energies. This is shown in Fig. 7 ▸. For some components, much higher doses (*e.g.* a factor of 100 times more) are required for phase contrast imaging at the higher energies. This is partly due to the fact that, at these higher energies above the water window, the main component of the cell (water) absorbs very strongly. Lipid and inner mitochondrial membranes would require the highest comparative fluence and dose at 4000 eV with starch granules the lowest and heterochromatin somewhere in between. However, if using objective zone plates with a low overall efficiency as an objective, the advantages of absorption contrast within the water window will decrease compared with lensless methods.

## 6. Discussion   

The analysis of fluence and dose requirements for imaging a protein in water shown in Figs. 2 ▸ and 3 ▸ contains a wide spread of values. When this spread is included for the cellular components along with the possibility of errors and variation in their compositions and densities, it might appear that the analysis provides poor guidance for X-ray imaging. However, the factors include different requirements, assumptions and uncertainties (defined here as imperfect information). Locating the same object in different fields of view is an example of different requirements. Whether a Rose criterion of 3 or 5 should be applied is an assumption about satisfactory statistics. An example of an uncertainty is the variation and errors in the composition and density of the cellular components, including the values for the density of water adopted, the composition of cytosol/nucleosol and the degree of compaction of heterochromatin. Many of the requirements, assumptions and uncertainties apply to both absorption and phase contrast imaging, including their energy dependence, so the comparison between these techniques is less dependent on the sample-dependent factors above. One other adjustment to be made is that for the overall attenuation length of the cell (Fig. 1 ▸) where, for example, only 0.135 of the signal would be transmitted at 520 eV for a 5 µm-thick cell with a water content of 70%.

At some energies there are only small differences between the refractive indices (real and/or imaginary) of particular cellular components and the cytoplasm/nucleoplasm. High fluence and dose requirements are then needed to detect the component. If, for example, starch granules can be seen using phase contrast at 4000 eV, up to ten times the fluence might be required to obtain the same contrast for other cellular components. Insufficient exposure might therefore be one of the reasons for the statement (Larabell & Nugent, 2010 ▸) concerning the difficulty of observing the rich array of organelles using CDI. Although it is theoretically possible, with sufficient statistics, to distinguish signals differing by very small amounts, it can place demands on the stability of the system. Being able to detect a 1% difference in contrast between two adjacent pixels on a detector would be very challenging. This situation might favour methods (*e.g.* Zernike phase contrast) which image contrast differences rather than the signal for each component separately, even though the statistics for the differences are similar. Small difference could still allow extended cellular components (*e.g.* membranes, fibres) to be identified as the contrast difference would extend over many pixels. However, it would be much more difficult to identify, for example, individual nucleosomes (10 nm in size) with such small contrast values.

The dose requirements for phase contrast increase by a factor of approximately two between 800 eV and 4000 eV (Fig. 5*b* ▸). The fluence requirements increase by a factor of approximately 30 between these two energies, thereby increasing exposure times. This would be particularly important for imaging by CDI or ptychography where the coherent portion of the total flux from the source would be quite low and, for many sources, both the total flux and coherent fraction decreases with increasing energy. This suggests that there is an advantage to operating at the lowest energy consistent with a low overall attenuation length.

The dose requirements for phase contrast at 4000 eV are between a factor of 22 (starch granule) and 450 (lipid droplet) higher than absorption contrast at 520 eV (Fig. 7 ▸). Mitochondrial membranes in absorption contrast at 522 eV would require a dose of 5.2 × 10^7^ Gy for 10 nm resolution whereas a dose of 1.5 × 10^10^ Gy would be required in phase contrast at 2000 eV. Howells *et al.* (2009 ▸) estimated a maximum tolerable dose of 1.0 × 10^8^ Gy nm^−1^ of resolution. The tolerable dose for 10 nm resolution of 10^9^ Gy is well within the requirement for imaging mitochondrial membranes by absorption contrast and just within the requirement for a protein in water using phase contrast, assuming the criteria adopted by Howells *et al.* (2009 ▸). At 2000 eV, the phase contrast between mitochondrial membrane and cytosol is much lower than that between protein and water and a much higher dose than 10^9^ Gy would be required to achieve 10 nm resolution. However, for 20 nm resolution, the required dose would be 16 times lower and the dose limit two times higher. Assuming the rather optimistic conditions used for these calculations (no phase errors, undefined total sample volume, Rose criterion of 5) it should therefore be possible to obtain a resolution of 20 nm.

The concentration of elements of medium atomic number such as phospho­rous, sulfur and potassium is quite low in the cellular components covered here. There appears to be little advantage in collecting data to exploit the absorption contrast of these elements in a similar manner to data collection in the water window. However, if carrying out scanning fluorescence microscopy the energy selected will be on the higher energy side of the absorption edges of interest. In this case the energy might not be optimized for other imaging methods (*e.g.* ptychography) if the data are collected simultaneously.

Matching the instrument (X-ray source, optics, detectors) to the requirements can assist in shortening data collection times and sometimes the dose using similar considerations to those analysed for crystallography (Nave, 2014 ▸). For example, in ptychography, the longitudinal coherence requirements can be quite modest and use of broader bandpass monochromators such as multilayers may offer some advantages in these circumstances. Optimization of the setup for high-throughput ptychography has been discussed in a recent paper by Jacobsen *et al.* (2017 ▸).

Full-field imaging using absorption contrast within the water window provides much more relaxed requirements in terms of both dose and fluence compared with methods based on phase contrast. Full-field microscopy in the water window would therefore appear to be the method of choice for thinner specimens provided a high-efficiency zone plate is used. At higher energies two major disadvantages are present. Firstly the high contrast between carbon (and nitro­gen) compared with oxygen is lost. Secondly there is increased energy deposition in the bulk of the sample (water) when operating on the high-energy side of the oxygen absorption edge. For thicker specimens with absorption contrast in the water window, unacceptable attenuation would be present together with increasing depth of focus problems if using a zone plate objective and these are the main reasons for using phase contrast methods at higher energy. These methods have provided good results for higher-density components such as starch grains (Diaz *et al.*, 2015 ▸), polyphosphate bodies (Diaz *et al.*, 2016 ▸) and bacterial nucleoids (Bartels *et al.*, 2012 ▸). Imaging lower-density components such as membranes is much harder at these energies because the signal depends on the square of the difference between the electron densities of the component of interest and the surrounding material. A recent paper (Villanueva-Perez *et al.*, 2018 ▸) calculated that imaging Compton scattered photons at incident X-ray energies around 64 keV would give a significant advantage compared with phase contrast microscopy in terms of deposited dose.

The calculations in this paper can of course be repeated as additional quantitative data becomes available from X-ray imaging and other techniques. For the case of X-ray imaging it would be useful if such data (*e.g.* refractive indices, absorption coefficients) could be obtained at several X-ray energies.

## Figures and Tables

**Table 1:** Values of the real and imaginary components of the refractive indices at selected energies

Energy (eV)	200		521.6264		4000	
	δ	β	δ	β	δ	β
Nucleosol	5.04 × 10^−3^	6.56 × 10^−4^	5.38 × 10^−4^	3.84 × 10^−5^	1.51 × 10^−5^	2.31 × 10^−7^
Heterochromatin	5.22 × 10^−3^	7.03 × 10^−4^	6.51 × 10^−4^	9.42 × 10^−5^	1.63 × 10^−5^	2.80 × 10^−7^
Cytosol	5.03 × 10^−3^	6.53 × 10^−4^	5.34 × 10^−4^	3.58 × 10^−5^	1.50 × 10^−5^	2.14 × 10^−7^
Inner mitochondrial membrane	5.07 × 10^−3^	6.28 × 10^−4^	7.33 × 10^−4^	1.38 × 10^−4^	1.64 × 10^−5^	2.21 × 10^−7^
Lipid droplet neutral core	3.89 × 10^−3^	2.58 × 10^−4^	7.52 × 10^−4^	1.68 × 10^−4^	1.34 × 10^−5^	9.04 × 10^−8^
Starch granule	5.95 × 10^−3^	7.02 × 10^−4^	7.62 × 10^−4^	1.04 × 10^−4^	1.87 × 10^−5^	2.32 × 10^−7^

**Table 2:** Approximate dimensions of some biological objects

Whole samples	
Pico-plankton	0.2–2 µm
*E. coli*	1.5 × 0.5 × 0.5 µm
Yeast	3.5 µm
Typical mammalian cell	10–30 µm
Typical plant cell	10–100 µm
Muscle myofibril	1–2 µm diameter

Components filaments (diameter)	
Microtubules	25 nm
Flagella, cilia	250 nm
Intermediate filaments	8–12 nm
Actin filaments	7 nm
Myosin	15 nm
Collagen fibril	50–200 nm
Collagen triple helix	1.5 nm
Chromatin fibre (*in vitro*)†	25–45 nm

Components planar (thickness)	
Membrane bilayer	3–7 nm
Yeast cell wall	100–200 nm
Perinuclear space	20–40 nm
Mitochondrial crista width	28 nm

Components globular	
Starch granule	2–100 µm
Nucleosome	11 nm
Nuclear pore complex (125mda)	145 × 145 × 80 nm
Ribosome	20–25 nm
Picornavirus (*e.g.* FMDV)	22–30 nm
Vaccinia virus	250 nm

† Although the putative 30 nm fibre for condensed chromatin has a dimension within the current range for X-ray imaging, analysis of cryo-electron microscopy images does not support the existence of 30 nm chromatin fibres in mitotic chromosomes *in situ* (Eltsov *et al.*, 2008 ▸; Maeshima *et al.*, 2014 ▸).

**Table 3:** Some cellular components with their compositions and densities

	Protein, Pr	Nucleic acid, Nu	Water, W	Lipid, L	Phospho­lipid, Ph	Potassium, K	Carbohydrate, Car
H (number)	50	51	2	98	79	0	10
C (number)	30	39	0	55	42	0	6
N (number)	9	15	0	0	1	0	0
O (number)	10	25	1	6	8	0	5
P (number)	0	4	0	0	1	0	0
S (number)	1	0	0	0	0	0	0
K (number)	0	0	0	0	0	1	0
Molecular mass	728.8	1253.8	18	855.4	757	39.1	162.1
PSV (ml g^−1^)	0.735	0.54	1	1.09	1.02	0.23	0.61
Density (g ml^−1^)	1.36	1.85	1	0.92	0.980	4.35	1.64

**Table 4:** Composition of each component (g l^−1^) and atomic ratios. For an example of the calculation, see Appendix *B* [App appb]. The resultant atomic ratios are given to more significant figures than are warranted by the uncertainty in the composition of the components. This is to enable others to repeat the calculations.

	Cytosol	Nucleosol	Hetero-chromatin	Mitochondrial inner membrane	Lipid droplet	Starch granule	Whole cell
Components (g l^−1^)							
Protein	60	60	267	556	0		177.2
Nucleic acid	30	30	133	0	0	0	88.58
Water	939.7	938.1	727.8	449.56	0	501.2	775.1
Lipid	0	0	0	0	920	0	22.1
Phospho­lipid	0	0	0	139	0	0	0
Potassium	0	7	18	0	0	0	11.07
Carbohydrate	0	0	0	0	0	817.7	33.22
Total	1030	1035	1146	1145	920	1319	1107

Atomic ratios							
H	109.7	109.6	104.6	104.6	105.4	106.1	106.5
C	3.40	3.40	15.13	15.13	59.15	30.27	12.70
N	1.10	1.10	4.89	4.89	0.00	0.00	3.25
O	53.63	53.54	46.75	46.75	6.45	53.07	48.44
P	0.10	0.10	0.42	0.42	0.00	0.00	0.28
S	0.08	0.08	0.37	0.37	0.00	0.00	0.24
K	0.00	0.18	0.46	0.46	0.00	0.00	0.28

**Table 5:** Summary of relevant parameters for cellular components obtained from literature

Component	LAC (µm^−1^) at 520 eV	Density (g ml^−1^)	Mass% H_2_O	Macromolecular concentration (g ml^−1^)	Mass ratios
Starch grains		1.29 ± 0.04^*a*^			
Cytoplasm		1.07 ± 0.012^*a*^			
Polyphosphate bodies		1.42 ± 0.09^*b*^			
Lipid droplets	0.7^*c*^, 0.75 ± 0.09^*d*^				
Vacuoles	0.22 ± 0.07^*c*^				
Heterochromatin	0.25–0.36^*c*^, 0.32 ± 0.02^*e*^				
Euchromatin	0.13–0.25^*c*^				
Nuclear chromatin yeast	0.26 ± 0.01^*c*^				
Nucleoids in bacteria		1.2–1.65^*f*^			
Nucleolus	0.33 ± 0.01^*c*^, 0.37 ± 0.04^*d*^				
Mitochondria	0.36 ± 0.02^*c*^, 0.45 ± 0.03^*d*^				
Average nuclei yeast/mammalian	0.26^*c*^				
Chloro­past	0.38 ± 0.09^*g*^				
Pyrenoid	0.44 ± 0.07^*g*^				
Nucleoplasm			75^*h*^		
Condensed Chromatin			65^*h*^	400^*i*^	
Whole cell			55–92.5^*j*^		
Mitochondria			60^*j*^		
Nucleolar fibrillar centres			83^*j*^		
Protein in cells				200–300^*k*^	
RNA in cells				75–150^*k*^	
Total protein and RNA				300–400^*k*^	
Nuclear chromatin protein:DNA					2:1^*l*^
Nucleolar chromatin protein:DNA					2.6:1^*l*^
Mitochondria outer membrane protein:lipid					1:1^*m*^
Mitochondria inner membrane protein:lipid					4:1^*m*^

^*a*^Diaz *et al.* (2015 ▸). ^*b*^Diaz *et al.* (2016 ▸). ^*c*^Do *et al.* (2015 ▸). ^*d*^Uchida *et al.* (2009 ▸). ^*e*^Le Gros *et al.* (2016 ▸). ^*f*^Bartels *et al.* (2012 ▸). ^*g*^Weiss *et al.* (2000 ▸). ^*h*^Nolin *et al.* (2012 ▸). ^*i*^Bohrmann *et al.* (1993 ▸). ^*j*^Nolin *et al.* (2013 ▸). ^*k*^Ellis (2001 ▸). ^*l*^Muramatsu & Onishi (1978 ▸). ^*m*^Hallermayer & Neupert (1974 ▸).

## Appendix A Supplementary tables

Refer to Tables 2 ▸, 3 ▸, 4 ▸ and 5 ▸, and the relating text in §1.4, §2 and §4.

## Appendix B Example calculation of atomic composition and density of heterochromatin

The volume occupied by each component has to be calculated in order to obtain the water content.

Protein H_50_C_30_N_9_O_10_S_1_, molecular mass 729.

267 g protein with a PSV = 0.735 ml g^−1^ occupies 196 ml.

Nucleic acid C_39_H_51_O_25_N_15_P_4_ corresponding to 50:50 AT:GC. Molecular weight 1254. 133 g nucleic acid with PSV = 0.54 ml g^−1^ occupies 72 ml.

Potassium (atomic weight 39) present at concentration of 462 mmol l^−1^ in condensed chromatin or 18 g l^−1^ (Nolin *et al.*, 2012 ▸, 2013 ▸). The volume of this could be ignored but, for completion, with an ionic radius of 0.15 nm this gives a partial specific volume of 0.23 ml g^−1^ and occupies 4.2 ml.

This leaves 728 ml water, *i.e.* 728 g. Total mass 1146 g. Mass fraction of water 728/1146 = 0.635. This is in reasonable agreement with the figure in the literature of 0.65 (Nolin *et al.*, 2012 ▸).

The atomic composition of chromatin is then calculated from the composition of the individual components, including water. For example, the carbon component (C_30_) of protein (molecular mass 729) will contribute 30 × 267/729 atoms and the nucleic acid component 30 × 133/1254 giving C_15.13_.

Calculations for other atoms give the formula H_105.6_C_15.1_N_4.9_O_46.8_P_0.4_S_0.4_K_0.5_. This formula is entered into the CXRO calculator along with a density of 1.146 g ml^−1^ to calculate the real and imaginary parts of the refractive index.
