# A comparison of absorption and phase contrast for X-ray imaging of biological cells. Erratum

**DOI:** 10.1107/S1600577519002601

**Published:** 2019-02-27

**Authors:** Colin Nave

**Affiliations:** a Diamond Light Source Ltd, Harwell Science and Innovation Campus, Didcot OX11 0DE, UK

**Keywords:** X-ray imaging, biological cells, dose and fluence requirements

## Abstract

An error in the calculation for X-ray absorption imaging has been identified in the paper by Nave (2018) [*J. Synchrotron Rad.***25**, 1490–1504]. The required fluence and dose in the paper are a factor of ten too low for this mode of imaging.

## Abstract 2

Corrections are given for the calculations of fluence and dose for X-ray imaging of biological cells by absorption in the paper by Nave (2018) [*J. Synchrotron Rad.***25**, 1490–1504].

The author regrets that an error in the calculations of the required fluence (and hence dose) for imaging by absorption was made in the paper by Nave (2018 ▸). This occurred because an incorrect number was applied when converting from the required number of photons incident on a pixel to a fluence (photons µm^−2^). The fluences and doses shown in Figs. 3 ▸ and 6 ▸ should all be increased by a factor of ten. As the error only occurred in the calculation for imaging by absorption, the dose ratios in Fig. 7 ▸ should be decreased by a factor of ten. These ratios are discussed in the text where the following corrections should be made:

Section 5.4: ‘For some components, much higher doses (*e.g.* a factor of 10–45 times more) are required for phase contrast imaging at the higher energies.’

Section 6, fourth paragraph: ‘The dose requirements for phase contrast at 4000 eV are between a factor of 2.2 (starch granule) and 45 (lipid droplet) higher than absorption contrast at 520 eV (Fig. 7 ▸). Mitochondrial membranes in absorption contrast at 522 eV would require a dose of 5.2 × 10^8^ Gy for 10 nm resolution whereas a dose of 1.5 × 10^10^ Gy would be required in phase contrast at 2000 eV.’

The benefits of operating in the water window for thin specimens are still present although to a lesser extent than given in the paper. The error was identified when comparing the calculations with those of Schneider (1998 ▸) in which optimized phase contrast (exploiting amplitude and phase contrast) in the water window gave the lowest required dose for thin specimens. The corrections given above are consistent with this conclusion.

## Figures and Tables

**Figure 3:**
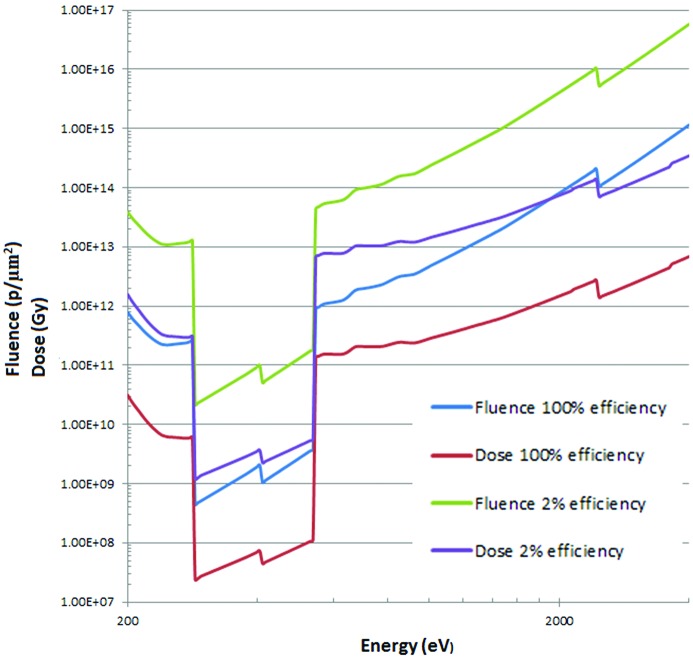
Fluence and dose for protein in water with absorption contrast. Rose criteria 5 with the dose distributed over a model cell (70% water). The fluence and dose at 2% efficiency follows the zone plate efficiency adopted by Huang *et al.* (2009 ▸).

**Figure 6:**
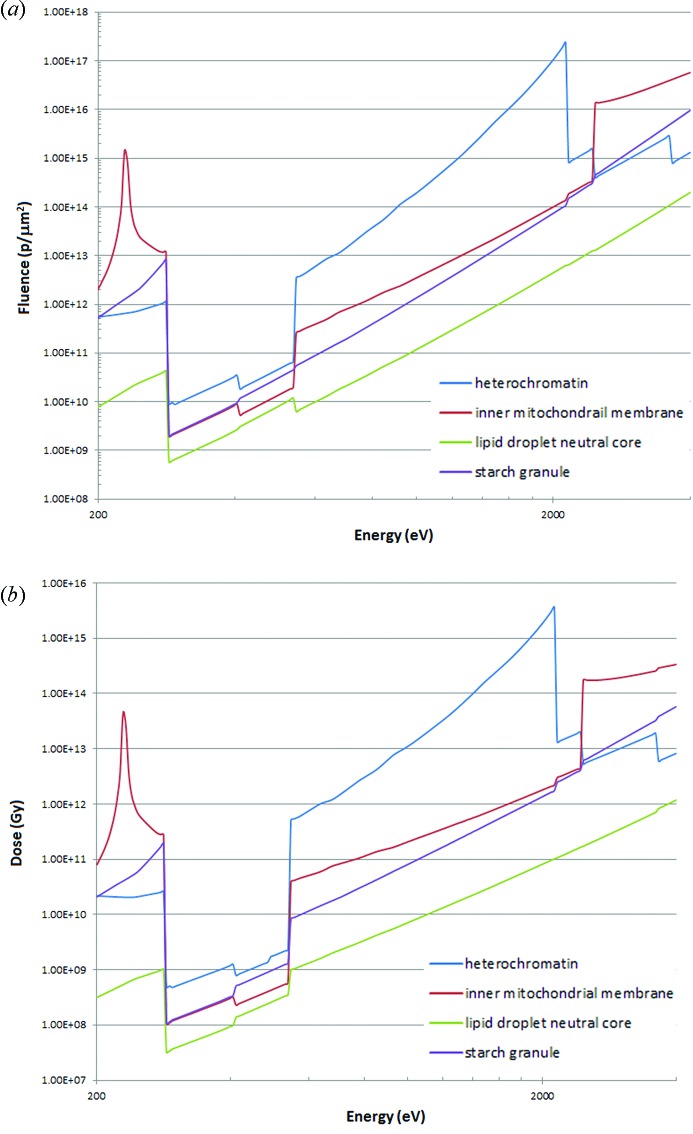
(*a*) Fluence requirements (absorption contrast, 10 nm resolution) for the four cellular components following the calculations for protein illustrated in Fig. 3, 100% efficiency. (*b*) Dose requirements following the calculations in Fig. 3, 100% efficiency.

**Figure 7:**
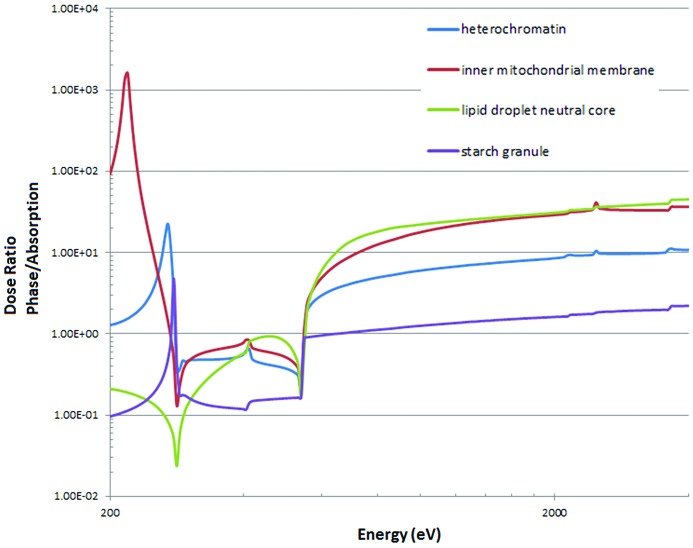
Comparison of the dose for phase contrast with absorption contrast at 520 eV. Obtained by dividing the values in Fig. 5(*b*) with the value at 520 eV in Fig. 6(*b*).
